# Simulation of an Asymmetric Photonic Structure Integrating Tamm Plasmon Polariton Modes and a Cavity Mode for Potential Urinary Glucose Sensing via Refractive Index Shifts

**DOI:** 10.3390/bios15100644

**Published:** 2025-09-29

**Authors:** Hung-Che Chou, Rashid G. Bikbaev, Ivan V. Timofeev, Mon-Juan Lee, Wei Lee

**Affiliations:** 1 Institute of Lighting and Energy Photonics, College of Photonics, National Yang Ming Chiao Tung University, Guiren Dist., Tainan 711010, Taiwan; roydinchow3@gmail.com; 2 Kirensky Institute of Physics, Federal Research Center KSC SB RAS, Krasnoyarsk 660036, Russia; bikbaev@iph.krasn.ru (R.G.B.); tiv@iph.krasn.ru (I.V.T.); 3 Institute of Engineering Physics and Radioelectronics, Siberian Federal University, Krasnoyarsk 660041, Russia; 4 Department of Chemical and Materials Engineering, National Kaohsiung University of Science and Technology, Sanmin Dist., Kaohsiung 807618, Taiwan; mjlee@mail.nsysu.edu.tw; 5 Institute of Imaging and Biomedical Photonics, College of Photonics, National Yang Ming Chiao Tung University, Guiren Dist., Tainan 711010, Taiwan

**Keywords:** Tamm plasmon polariton, optical sensor, refractive index sensing, cavity mode, diabetes sensing

## Abstract

Diabetes has become a global health challenge, driving the demand for innovative, non-invasive diagnostic technologies to improve glucose monitoring. Urinary glucose concentration, a reliable indicator of metabolic changes, provides a practical alternative for frequent monitoring without the discomfort of invasive methods. In this simulation-based study, we propose a novel asymmetric photonic structure that integrates Tamm plasmon polariton (TPP) modes and a cavity mode for high-precision refractive index sensing, with a conceptual focus on the potential detection of urinary glucose. The structure supports three distinct resonance modes, each with unique field localization. Both the TPP modes, confined at the metallic–dielectric interfaces, serve as stable references whose wavelengths are unaffected by refractive-index variations in human urine, whereas the cavity mode exhibits a redshift with increasing refractive index, enabling high responsiveness to analyte changes. The evaluation of sensing performance employs a sensitivity formulation that leverages either TPP mode as a reference and the cavity mode as a probe, thereby achieving dependable measurement and spectral stability. The optimized design achieves a sensitivity of 693 nm·RIU^−1^ and a maximum figure of merit of 935 RIU^−1^, indicating high detection resolution and spectral sharpness. The device allows both reflectance and transmittance measurements to ensure enhanced versatility. Moreover, the coupling between TPP and cavity modes demonstrates hybrid resonance, empowering applications such as polarization-sensitive or angle-dependent filtering. The figure of merit is analyzed further, considering resonance wavelength shifts and spectral sharpness, thus manifesting the structure’s robustness. Although this study does not provide experimental data such as calibration curves, recovery rates, or specificity validation, the proposed structure offers a promising conceptual framework for refractive index-based biosensing in human urine. The findings position the structure as a versatile platform for advanced photonic systems, offering precision, tunability, and multifunctionality beyond the demonstrated optical sensing capabilities.

## 1. Introduction

The rising incidence of diabetes has underscored the imperative for timely diagnosis and effective monitoring to mitigate the associated health risks [1]. Among the various biomarkers, the refractive index of urine associated with glucose concentration is a crucial indicator for the diagnosis of diabetes and the evaluation of metabolic health. When blood glucose levels exceed the physiological renal threshold, typically in the range of 0–15 mg·dL^−1^, the kidneys excrete excess glucose into the urine [2]. This phenomenon modulates the refractive index of urine and provides a basis for simulation-driven optical analysis under glucose-relevant conditions. While acknowledging that urinary glucose testing does not yield precise blood glucose measurements, it is widely regarded as a significant advance over traditional invasive methods. This approach is noninvasive and straight-forward to perform, making it particularly beneficial for individuals with limited venous access or those requiring frequent monitoring [3,4,5]. These advantages highlight the necessity for advancing techniques to analyze refractive index changes in urine associated with glucose concentration with greater sensitivity and accuracy. Developing innovative, high-performance refractive index-based biosensing platforms has become a critical research area to address these needs. A measurable correlation between glucose concentration and refractive index in human urine has been experimentally demonstrated, supporting the feasibility of simulation-driven optical models for noninvasive glucose monitoring [6]. The goal is to create reliable, efficient, and patient-friendly solutions for diabetes management by integrating optical sensing principles and leveraging modern advancements.

In recent years, there has been significant progress in the development of innovative glucose sensing technologies, which have the potential to deliver trustworthy and patient-centered approaches to diabetes care and management. Highly sensitive sensors for noninvasive glucose detection employ state-of-the-art methods, including cutting-edge photonic measurement techniques [7,8,9], noninvasive blood glucose monitoring systems [10,11], and blood glucose monitoring in the terahertz-frequency range [12]. Integration of these sensors with portable and user-friendly platforms has promoted their applicability in point-of-care testing and home-based monitoring [13,14]. These advancements also provide a foundation for theoretical studies, such as the one presented herein, which aim to support future biosensor design.

Among the aforementioned technologies, optical sensors have emerged as a particularly promising platform and a key focus in sensing research. As an important subclass of sensors, optical sensors offer unique advantages, including label-free detection, real-time monitoring, and minimal dependence on environmental conditions [15,16,17,18]. Within the domain of optical sensing, surface plasmon polariton (SPP) [19] and Tamm plasmon polariton (TPP) [20] technologies have demonstrated remarkable potential for attaining high sensitivity in biomedical diagnostics, particularly in applications relevant to refractive index-based glucose sensing in biofluids such as urine. SPP is a well-established technique that relies on the excitation of surface plasmons (i.e., oscillations of conduction electrons at the interface between metals and dielectrics) under specific conditions, resulting in a substantial reduction in reflectivity [21]. However, the incorporation of SPP systems into compact devices is hindered by the requirement of prism or grating couplers and precise incident angles [22,23,24,25]. Moreover, SPP does not exist for transverse electric (TE) polarization, but only for transverse magnetic (TM) polarization.

In contrast, TPPs grant a promising alternative by addressing many of SPR’s limitations. The TPPs can be defined as localized optical states confined at the interface between a metallic layer and a distributed Bragg reflector (DBR) [26]. The photonic bandgap (PBG) of the DBR, in conjunction with electromagnetic attenuation in the metal, imprisons the TPPs at the interface [27]. In contradistinction to SPP, TPPs can be excited under normal and oblique incidences using either TE or TM polarizations without the necessity for additional coupling elements such as a prism or grating [28]. These benefits render TPPs highly suitable for sensing applications, providing promoted sensitivity, improved design flexibility, and simplified integration. In addition to the merits of TPPs, cavity modes in metal-dielectric-metal structures represent a promising approach to optical sensing [29]. The formation of these modes is attributed to the constructive interference of light confined within the cavity, which is sandwiched between two metallic layers [30]. The formation of a standing wave resonance is contingent upon the thickness of the cavity satisfying a specific condition. Compared with TPPs, cavity modes permit a greater degree of tunability through geometric and material modifications, enabling precise control over resonance frequencies [31]. The unique attributes of cavity modes, such as their scalability and compatibility with miniaturized systems, make them highly attractive for a wide range of sensing purposes, including biomedical diagnostics and environmental monitoring [32,33].

In this study, we propose a novel optical sensing platform designed with potential applicability to refractive index-based detection scenarios, such as monitoring glucose concentration variations in biofluids including urine. The architecture incorporates two strategically engineered DBRs and two adjacent metallic layers to excite TPPs [34], achieving high sensitivity and strong field localization to enhance light-matter interactions [35]. This innovative design offers not only exceptional sensitivity but also greater flexibility for structural optimization and device integration. Through comprehensive theoretical analysis and numerical simulations, we investigate the influence of some critical design parameters, such as DBR layer thickness and metallic material, on sensor performance metrics, including the quality factor (*Q*), guidance factor (*G*), sensitivity (*S*), and figure of merit (FoM) [36,37,38]. Compared with existing TPP-based designs and even some SPP-based sensors, our devised sensing structure demonstrates enhanced sensitivity and spectral accuracy, enabled by resonance mode tuning and structural optimization [39,40]. This work provides a theoretical foundation for refractive index-based glucose monitoring in biomedical applications, highlighting the potential of the proposed structure to inform future sensor development.

## 2. Device Configuration and Resonance Analysis

**Figure 1:**
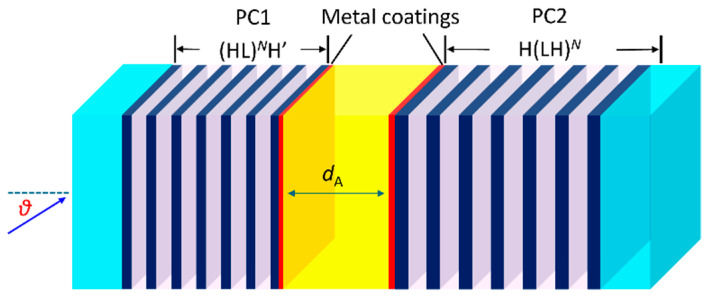
Schematic of the designed asymmetric photonic structure with the number of periods of the alternating dielectric layers, *N* = 6. The structure consists of two DBRs sandwiching a cavity with two interfacing metallic (e.g., Ag) films. The analyte layer is positioned at the center of the structure to fill the cavity, with a thickness of *d*_A_ = 1 μm.

As illustrated in Figure 1, the proposed asymmetric sensor utilizes the combined principles of photonic crystal (PC) and TPP to achieve exceptional sensitivity and selectivity for optical sensing applications. The framework consists of two distinct one-dimensional (1D) PCs (PC1 and PC2) that are arranged on both sides of a central analyte cavity. At the interfaces between the cavity and the PCs, thin metallic layers are introduced to support the excitation of TPP modes, which play a critical role in enhancing the platform’s optical response. Each 1D PC on a glass substrate is constituted by alternating high-index (H) TiO_2_ and low-index (L) SiO_2_ dielectric layers to form a DBR. These DBRs are engineered to provide high reflectivity and create a PBG that ensures strong optical confinement within the structure. The H and L layers are designed with quarter-wavelength (*λ*/4) optical thicknesses d at 760 nm (*d*_H1_ = 75 nm and *d*_L1_ = 130 nm) for PC1 and 1000 nm (*d*_H2_ = 100 nm and *d*_L2_ = 150 nm) for PC2, assuring optimal performance across the operating wavelength range. Notably, the H dielectric layer adjacent to the metallic coating within PC1 is specifically designed with a reduced thickness of 65 nm (denoted as H’ in Figure 1), in contrast to the other H layers set at 75 nm. This localized reduction in thickness enhances the confinement of the electromagnetic field, thereby optimizing the localization of the field distribution. Base on the dispersion parameters for TiO_2_ and SiO_2_ obtained from the material library of COMSOL Multiphysics 6.0 (Stockholm, Sweden), the refractive indices *n* of these dielectric layers are wavelength-dependent as described by the following Cauchy dispersion relations:

(1) $$n_{{\mathrm{TiO}}_{2}}=2.39513+\frac{0.03471}{\lambda^{2}}-\frac{0.00835}{\lambda^{4}}$$

(2) $$n_{{\mathrm{SiO}}_{2}}=1.46705+\frac{0.00364}{\lambda^{2}}-\frac{3.049\times 10^{-6}}{\lambda^{4}}$$

where *λ* is in μm, and *n*_TiO2_ and *n*_SiO2_ represent the refractive indices of the high- and low-index materials, respectively. Both Cauchy equations presented above account for the dispersive properties of the dielectric layers with high coefficients of determination (*R*^2^ = 1 for 0.30 μm ≤ *λ* ≤ 1.25 μm), which are critical for achieving precise control over the PBG and optimizing light confinement within the structure. The metallic layers (say, of Ag) of *d*_M_ = 30 nm in thickness at the interfaces sustain TPP modes, enabling hybrid resonance phenomena when coupled with the optical cavity. The thickness (*d*_A_) and refractive index (*n*_a_) of the central analyte layer can be tuned to allow precise modulation of the sensor’s optical response. The hybridization of cavity and TPP modes elevates the device’s sensitivity and selectivity, making it a prospective platform for label-free biosensing applications. The modeling of the refractive indices of the dielectric layers, as described by the above equations, is pivotal in optimizing the PBG and TPP-cavity coupling, accordingly ensuring the sensor’s performance across a broad wavelength range.

To achieve resonance within the structure, the eigenfrequency (*ω*) of the system is governed by the phase-matching condition:

(3) $$\varphi_{1}+\varphi_{2}+\varphi_{A}=2\pi m$$

where *φ*_1_ = arg (*r*_1_), *φ*_2_ = arg (*r*_2_) and *r*_1_, *r*_2_ represent the phase shifts in the reflected waves and the amplitude reflection coefficients at the PC1-Ag and Ag-PC2 boundaries, respectively, *φ*_A_ = 2*n*_a_*d*_A_*ω*/*c* accounts for the phase change for wave propagation through the analyte layer, and m is an integer representing the mode order. Note that each phase *φ*_1_, *φ*_2_, and *φ*_A_ is dependent on the refractive index of the analyte medium. The phase-matching condition expressed by Equation (1) is critical for the formation of localized resonance modes, such as the first TPP (TPP1), cavity mode, and the second TPP (TPP2). These modes arise from the precise interplay between the phase shifts at the metallic-dielectric interfaces and the optical path length within the analyte layer. Specifically, TPP1 and TPP2 are predominantly localized at the metal–dielectric interfaces, whereas the cavity mode exhibits strong field confinement within the analyte layer. The interrelation between these resonance modes fundamentally determines the optical behavior of the device, including its reflection, transmission, and absorption spectra. Moreover, the sensitivity and tunability of the configured device rely on the unambiguous control of the phase-matching condition, making it a robust platform for label-free sensing applications and optical filtering in the targeted spectral range. In the proposed structure the light leaks out from the filled cavity with refractive index *n*_a_. As a result, the eigenfrequency gives wavenumber *k* = *ω*/2*πc* measured in inverse microns that can be explicitly derived from Equation (1) as:

(4) $$k=\frac{2\pi m-\arg(r_{1}(n_{a}))-\arg(r_{2}(n_{a}))}{4\pi n_{a}d_{a}}$$

The solution of Equation (2) enables the determination of the frequency of the localized state in relation to the refractive index of the analyte, *n*_a_. This equation demonstrates how the resonance frequency is contingent on *n*_a_ and *d*_A_, in addition to the phase contributions from the metallic interfaces. These relationships are crucial for understanding and optimizing the hybridization of TPP and cavity modes.

The reflectance, transmittance and absorptance spectra of the structure can be calculated using the transfer-matrix method [41]. The transfer matrix of the entire structure that relates the amplitudes of the incident and transmitted waves is a product of 2 × 2 matrices:

(5) $$M=T_{01}T_{02}\ldots T_{N-1,N}T_{N,S}$$

where the transfer matrix is:

(6) $$T_{n-1,n}=\frac{1}{2}\left(\begin{array}{cc} (1+h)e^{-i\alpha_{n}\gamma_{n}} & (1-h)e^{i\alpha_{n}\gamma_{n}}\\ (1-h)e^{-i\alpha_{n}\gamma_{n}} & (1+h)e^{i\alpha_{n}\gamma_{n}} \end{array}\right)$$

Here, *h* = (*ε_n_*/*ε_n_*_-1_)^1/2^, *ε*(*n*) is the permittivity of the *n*-th layer, *α* = (*ω*/*c*)*ε*(*n*)^1/2^, *ω* is the wave frequency, *c* is the speed of light, and *γ_n_ = z_n −_ z_n__−_*_1_ is the layer thickness, where *n* = 1, 2,…, *N* and *z_n_* is the coordinate of the interface between the *n*-th layer and the (*n* + 1) layer adjacent from the right (*γ_N_*_+1_ = 0). The transfer matrix for the orthogonally polarized wave is obtained from Equation (6) by substituting (*ε_n_*_−1_/*ε_n_*)^1/2^ for *h*. The transmittance, reflectance and absorbance are determined as:

(7) $$T\left(\omega \right)=\frac{1}{{\left|{\hat{M}}_{11}\right|}^{2}},R\left(\omega \right)=\frac{{\left|{\hat{M}}_{11}\right|}^{2}}{{\left|{\hat{M}}_{21}\right|}^{2}},A(\omega )=1-T(\omega )-R(\omega )$$

where *M*_11_ and *M*_21_ are the elements of matrix *M*.

## 3. Results and Discussion

**Figure 2:**
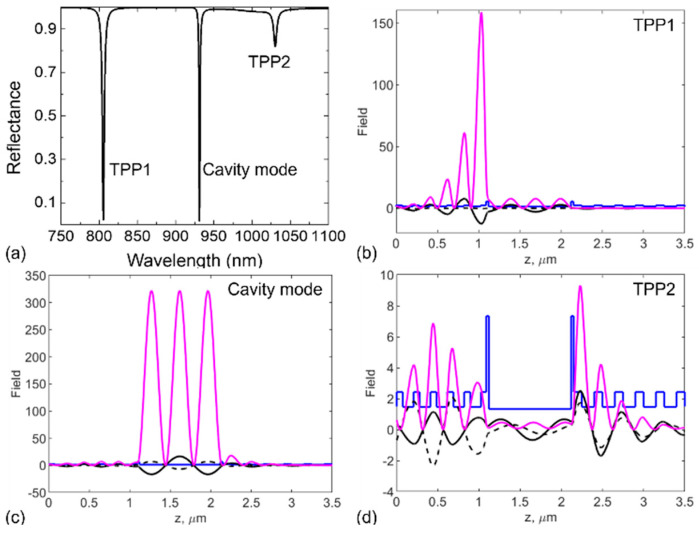
Reflectance spectrum and electric-field distributions of the photonic structure that contains two Ag films enclosing an analyte (normal human urine) of *n*_a_ = 1.335 calculated by transfer-matrix method and verified by the software COMSOL Multiphysics 6.0 (Stockholm, Sweden). (**a**) Reflectance spectrum of the structure showing three resonance modes. (**b**–**d**) Field intensity distributions (magenta curves) for TPP1, cavity, and TPP2 modes, respectively. The blue curves represent the spatial variations in refractive index, and the black solid and dashed lines show the spatially distributed real and imaginary parts of the electric field, respectively.

As depicted in Figure 2a, the reflectance spectrum of the designed device structure demonstrates three distinct resonance signals within the wavelength range of 750–1100 nm. These spectral lines correspond to two TPP modes and a cavity mode in between. The detailed electric-field distributions of these modes are illustrated in Figure 2b–d, high-lighting their unique field-localization characteristics. The first resonance mode, TPP1, as shown in Figure 2b, exhibits strong field confinement at the metallic-dielectric interface. This mode, a hallmark of TPP excitation, results from electro-magnetic field localization at the interface between the metal and the DBR. The high reflectivity of the DBR and the negative permittivity of the metal combine to facilitate the formation of a confined TPP mode with requirement of the phase-matching condition.

(8) $$\varphi_{\mathrm{PC}1,2}+\varphi_{\mathrm{Metal}}=2\pi m_{1,2}$$

The PBG properties of PC1 further enhance optical confinement near the metallic interface, leading to a highly localized electric field. Conversely, the cavity mode (Figure 2c) shows significant field enhancement within the central layer. This mode manifests a high degree of sensitivity to variations in the analyte’s refractive index. This sensitivity renders the cavity mode particularly well-suited for label-free sensing applications, where the detection of small changes in analyte properties is imperative. Unlike the TPP1 mode, the cavity mode exhibits higher transmittance due to its localization within low-loss dielectric regions, allowing more energy to propagate through the structure. The third resonance mode, TPP2, shown in Figure 2d, exhibits a field distribution localized at the PC–metal interface, similar in spatial confinement to TPP1, but occurs at a longer wavelength. This redshift is primarily caused by the difference in optical thickness between PC1 and PC2, which modulates the interaction between the TPP mode and the cavity resonance. The spatial overlap and coupling between the cavity mode and the TPP modes illustrate the hybrid nature of these resonances, significantly increasing the spectral sensitivity of the device. Such optical functionality of the proposed structure is desired in that the interplay between TPP and cavity resonances allows precise control over mode localization and resonance wavelengths. The hybridization not only consolidates electric field confinement and spectral selectivity but also facilitates the tunability of the sensor’s optical response. These features make the device a robust platform for label-free and highly sensitive analyte detection.

**Figure 3:**
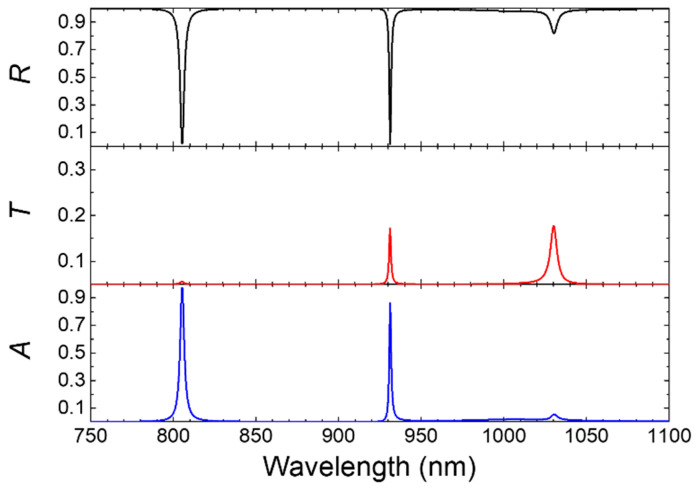
Reflectance (*R*), transmittance (*T*), and absorptance (*A*) spectra of the photonic structure in the near-infrared range. The spectra highlight three distinct resonance modes at 805 (TPP1, Figure 2b), 931 (Cavity mode, Figure 2c), and 1030 nm (TPP2, Figure 2d), with complementary features observed between reflectance and transmittance, and enhanced absorption at resonance wavelengths for an analyte with *n*_a_ = 1.335. The PBG of initial PC1 and PC2 are overlapping the same way as in Figure 2 in Reference [43].

Figure 3 presents the reflectance (*R*), transmittance (*T*), and absorptance (*A*) spectra of the proposed asymmetric photonic structure, providing a detailed understanding of the device’s optical behavior across the wavelengths spanning from 750 to 1100 nm. The three resonance modes correspond to distinctive optical signatures, underlining their particular functions in spectral control and sensing applications. The reflection spectrum displays prominent dips at the resonance wavelengths, indicative of efficient light coupling into the structure. The transmission spectrum furnishes a complementary perspective, exhibiting minimal transmittance for TPP1 (~0%) and moderate values for the cavity mode (~20%) and TPP2 (~20%). The absorptance spectrum unravels peaks at the three resonance wavelengths, signifying strong absorption especially at both 805 and 931 nm. This absorption primarily originates from the intrinsic optical loss of the metallic layer, which is characterized by the non-zero imaginary part (*k*) of its complex refractive index, as reported by Johnson and Christy [42]. The combination of spectral characteristics in reflection, transmission, and absorption showcases the device’s versatility for optical filtering and analyte detection.

The TPP1 mode is characterized by its strong localization at the metallic–dielectric interface and negligible transmission. This renders TPP1 an optimal reference line at 805 nm in reflectance spectra additionally due to its stability or minimal sensitivity to variations in refractive index of the analyte layer (to be discussed later). Conversely, the cavity mode, with its substantial field enhancement within the analyte layer, exhibits a balance between reflectance and transmittance. It is susceptible to changes in *n*_a_ to enable precise analyte detection, which is critical for label-free sensing applications. The TPP2 mode, occurring at a longer wavelength at 1030 nm, displays moderate transmission. Likewise, its stable spectral-line properties make TPP2 another suitable reference in the transmission spectrum for sensing. The functional duality of TPP1 and TPP2 as references in spectral reflectance and transmittance, respectively, allows the outlined structure to achieve self-referenced sensing capability.

**Figure 4:**
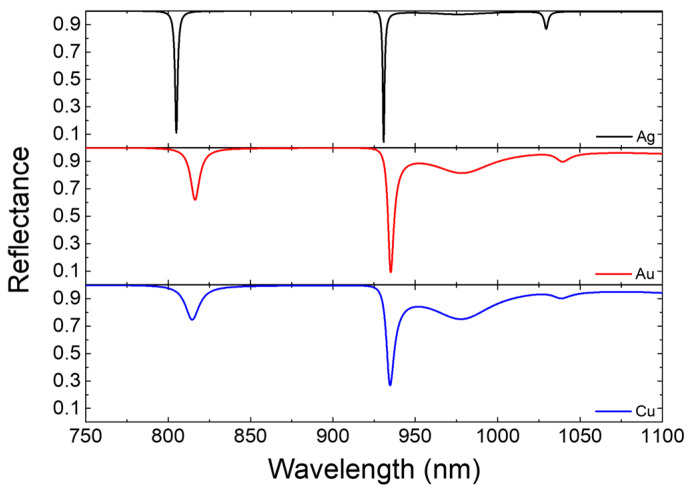
Comparative reflectance spectra of the proposed structure containing three different metallic materials (Ag, Au, and Cu) under equal layer thicknesses and identical structure parameters for PC1-M-A-M-PC2. The spectra accentuate the influence of the metallic layers on the resonance quality and reflection performance. *n*_a_ = 1.335.

Figure 4 shows the reflectance spectra of three photonic structures, each consisting of a different metallic material (Ag, Au, and Cu from top to bottom). Outside the three wavelengths of resonance, the use of silver consistently maintains good contrast, whereas the choices of both gold and copper for the metal films worsen the contrast, bringing about spurious signals around 1000 nm and compromised spectral clarity. These spurious signals hinder accurate signal interpretation and reduce the overall signal-to-noise ratio, posing a crucial challenge for the functionality of the sensing device. The superior optical performance of Ag can be attributed to its higher electrical conductivity and lower intrinsic absorption losses in comparison with those of Au and Cu. These material properties enable Ag to suppress non-resonance energy dissipation while preserving high reflectance outside resonance regions. This critical feature enhances photonic devices by ensuring consistent optical performance across a broad spectrum, boosting reliability, sensitivity, and precision.

To further quantify the spectral performance, two key metrics are employed: the quality factor (*Q*-factor) and guidance factor (*G*-factor). The *Q*-factor is conventionally expressed as

(9) $$Q=\frac{\lambda }{\mathrm{FWHM}}$$

where *λ* is the wavelength of resonance excited by TPP or cavity, and FWHM is the full width at half maximum or minimum of the resonance mode. It quantifies the sharpness of the resonance and indicates the spectral resolution. Meanwhile, we introduce the dimensional *G*-factor in μm^−1^, defined as the following equation:

(10) $$G=\frac{1-R}{\mathrm{FWHM}}$$

where *R* is the reflectance of a TPP or cavity mode. This factor captures a balance between signal strength and FWHM, providing a comprehensive measure of both resonance efficiency and energy confinement. In this study, the optimal thicknesses of the high- and low-refractive-index dielectric layers are first determined (in accordance with the unitless *Q*-factor) and then fixed, while the metal film thickness is further optimized to attain the best signal response. During the optimization process, spectral discrepancies caused by variations in the metal film thickness result in relatively minor changes in the *Q*-factor, whereas the *G*-factor exhibits more substantial differences. This suggests that the *G*-factor is a more effective indicator for assessing structural variations in the metal layers. We relied on the *G*-factor to determine the optimal metal film thickness (*d*_M_) of 30 nm. By using these indicators, we obtained the comparative values for Ag, Au, and Cu as displayed in Table 1. One can see that silver gives the highest *Q*- and *G*-factors among the three common metals, marking superior resonance sharpness and signal strength. This performance characterizes silver’s ability to maintain tightly confined and high-quality optical resonances, making it the most effectual metal for generating TPP modes in this study.

Optimizing material selection to leverage the properties of Ag allows for promoted spectral discrimination and operational stability, as well as for the development of next-generation optical sensors with high precision and reliability. The integration of a high *Q*-factor, exceptional *G*-factor, and consistent reflectance ensures superior spectral clarity, reduces noise, and maximizes sensitivity. These attributes not only improve sensing performance but also enable broader applications in advanced photonic systems for optical communications, environmental monitoring, and biomedical diagnostics. The resonance signals disclose resonance conditions within the target wavelength range, featuring strong optical coupling at specific wavelengths. The absorptance spectrum further reveals the fraction of light energy absorbed by the device at these resonance modes, rendering a comprehensive characterization of the optical behavior and energy distribution. These results demonstrate the interplay between reflection, transmission, and absorption in the formulated framework.

**Figure 5:**
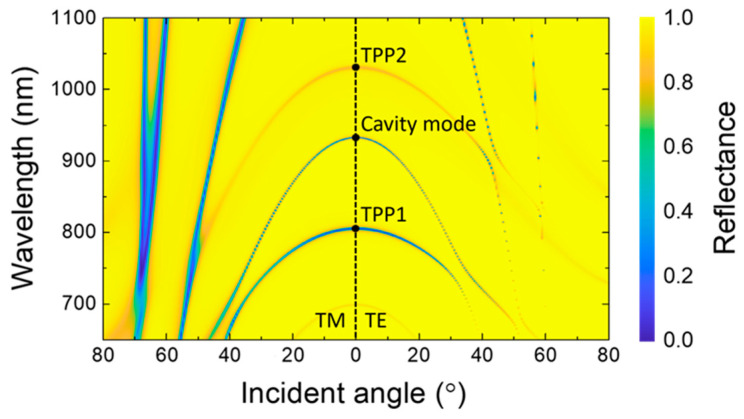
Angular-dependent reflectance spectra of the proposed structure for TM- and TE-polarized waves. The spectra illustrate the reflective behavior of the device as a function of the incident angle, highlighting angularly tunable resonance modes and their distinct responses for TM and TE polarizations. The figure delineates the evolution of reflectance over a range of incident angles *θ*, emphasizing the interplay between resonance modes under different polarization conditions.

As delineated in Figure 5, the reflectance spectrum of the proposed structure is dependent on the incident angle, with the emergence of distinct spectral bands due to angular shifts in resonance modes. It is evident that, as the incident angle increases, the resonance modes undergo notable shifts toward shorter wavelengths, implying strong angular dispersion. These shifts arise from the coupling between the TPP modes and the cavity mode, leading to the formation of hybrid resonance states at specific angles. To facilitate comparative observation, Figure 5 is configured so that the left and right halves, corresponding to positive angles of incidence, outlines the TM and TE reflection behaviors, respectively. The reflection characteristics of the photonic structure for TM- and TE-polarized waves divulge distinct polarization-dependent natures, with a distinguished coupling occurring at an incident angle of *θ* = 35° for TE-polarized waves. Here the cavity mode couples with the TPP1 mode, giving rise to a hybrid TPP-cavity mode. This hybridization is characterized by enhanced optical localization and improved spectral sensitivity, underscoring the unique interaction between these two resonances. The absence of the Brewster effect, typically observed for TE-polarized waves at dielectric interfaces, further highlights the role of the metallic layer in the structure. The inclusion of the metallic layers disrupts the conditions necessary for Brewster’s angle to occur, while simultaneously enabling robust coupling between the TPP and cavity modes. The angular tunability shown in Figure 5 enables precise control over resonance conditions, as seen at *θ* = 35°, where TPP1 couples with the cavity mode to form a hybrid resonance. This interaction enhances spectral selectivity and confinement, offering flexibility for applications such as angular-dependent filtering and high-precision sensing. These findings emphasize the critical role of the angle of incidence in modulating the optical response of the structure. By utilizing the angular dependence of hybrid resonances, this asymmetric structure offers valuable insights for the design of photonic systems that are optimized for tunable, high wavelength and polarization-sensitive applications.

**Figure 6:**
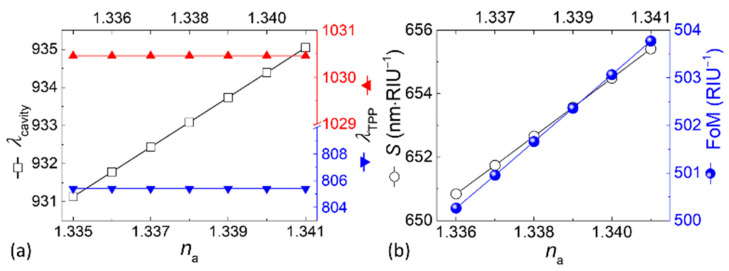
Refractive-index-dependent resonance wavelength shifts and performance analysis of the proposed structure. (**a**) Resonance wavelengths of the three modes and (**b**) reflection- and transmission-based sensitivity (*S*) and figure of merit (FoM) analysis. Both *S* and FoM in the ordinates in (**b**) enable a comprehensive evaluation of the biosensor’s sensing capabilities. RIU stands for Refractive Index Unit in the units.

The responsiveness of the asymmetric PC1-M-A-M-PC2 photonic structure to varying *n*_a_ is exhibited in Figure 6, where *n*_a_ ranges from 1.335 [5] at glucose concentrations of 0–15 mg·dL^−1^ in human urine to 1.341 at 5 g·dL^−1^, representing a theoretical model of refractive index variations associated with physiological and elevated glucose levels. These values can be used to evaluate the structure’s performance under realistic sensing conditions. As shown in Figure 6a, the TPP modes display minimal sensitivity to variations in *n*_a_, thus ensuring reliability as a reference for calibration in reflectance-based sensing applications. Conversely, the cavity mode exhibits significant shifts in its resonance wavelength in response to changing *n*_a_, highlighting its capacity for high-sensitivity detection. The collective manifestation of these characteristics is indicative of the devised structure’s capacity to facilitate both stable reference modes and highly responsive sensing capabilities. The sensitivity of the system can be determined using the following equation:

(11) $$S=\left|\frac{\Delta \lambda_{\mathrm{cavity}}-\Delta \lambda_{\mathrm{TPP}}}{n_{a}-1.335}\right|$$

where *λ*_TPP_ and *λ*_cavity_ in nanometers are the resonance wavelengths of the TPP1 or TPP2 and cavity modes, respectively, and 1.335 in the denominator is the value as a reference corresponding to the refractive index of normal human urine. This study proposes an alternative definition of *S*, diverging from the conventional definition typically expressed as *S* = Δ*λ*/Δ*n*. Our modified definition accounts for any minute (multilayer) dimensional tolerance in the manufacturing of the photonic devices. Ideally, *λ*_TPP_ is constant (805.397 nm for TPP1 and 1030.456 nm for TPP2) to allow Δ*λ*_TPP_ to vanish and Δ*λ*_cavity_ to become *λ*_cavity_ − 931.129 nm. The cavity mode, which undergoes a shift in wavelength in response to an alteration in the analyte’s refractive index, functions as the probe. The system’s ability to detect subtle changes in refractive index, such as those modeled to represent variations in glucose concentration in urine, is enhanced by this dual-reference design. In addition to its exceptional performance in reflection spectra, the proposed design manifests significant potential for applications in transmission-based sensing systems. The integration of either stable reference and highly sensitive probe modes renders precise refractive-index sensing capabilities across reflectance and transmittance spectra. This versatility enhances the applicability of the device in optical sensing technologies, particularly in scenarios requiring simultaneous or independent analysis of reflection and transmission characteristics within the target wavelength region.

The figure of merit (FoM) can be formulated as

(12) $$\mathrm{FoM}=\frac{S}{\mathrm{FWHM}}$$

**Figure 7:**
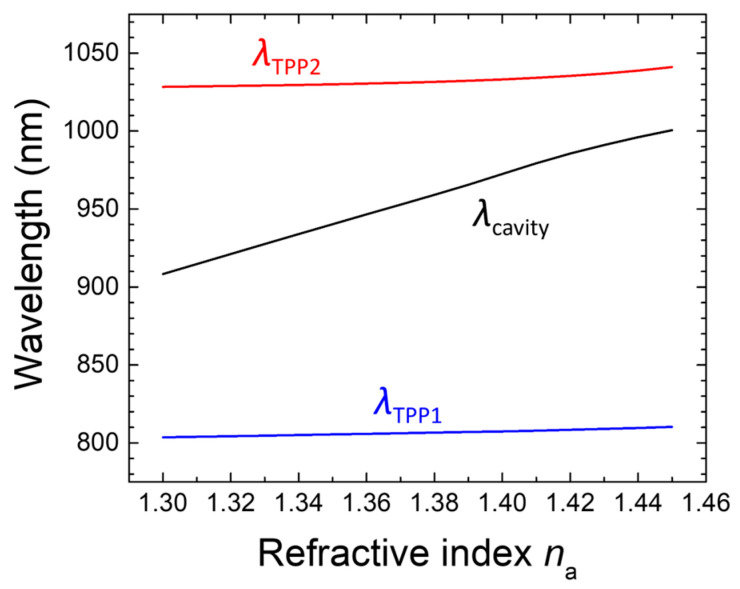
Dependence of the resonance wavelengths of three different modes on the refractive index of the analyte layer.

The incorporation of both sensitivity *S* and spectral sharpness represented by FWHM into FoM provides an insightful assessment of the device’s sensing performance. This parameter in RIU^−1^ is augmented by a narrow resonance peak of the cavity mode, essential for reducing noise and enhancing detection accuracy. The ability to balance sensitivity and resolution through FoM reflects the structural design’s optimization, which enables reliable operation across varying analyte conditions. As presented in Table 2, which shows the literature-derived refractive-index values of human urine with different glucose concentrations along with the corresponding simulated sensitivities and FoMs, the combination of these two metrics ensures that the device achieves both high sensitivity and robust spectral performance. Additionally, both *S* and FoM vary linearly with *n*_a_ (Figure 6b), demonstrating the consistency of the structure’s optical response. Beyond the application for diagnosis of diabetes as described above, the structured device performs reliably over a wider range for general refractive-index sensing purposes, offering adaptability to diverse sensing environments for high-precision applications in a variety of fields such as biosensing, chemical sensing, materials characterization, optical diagnostics, environmental monitoring, food quality control, and medical diagnostics. For example, Figure 7 suggests that the proposed structure is not limited to simulated detection scenarios involving glucose concentration in urine but is also potentially applicable to other sensing scenarios, such as analyzing blood composition or classifying cancer cell types [44,45,46].

**Figure 8:**
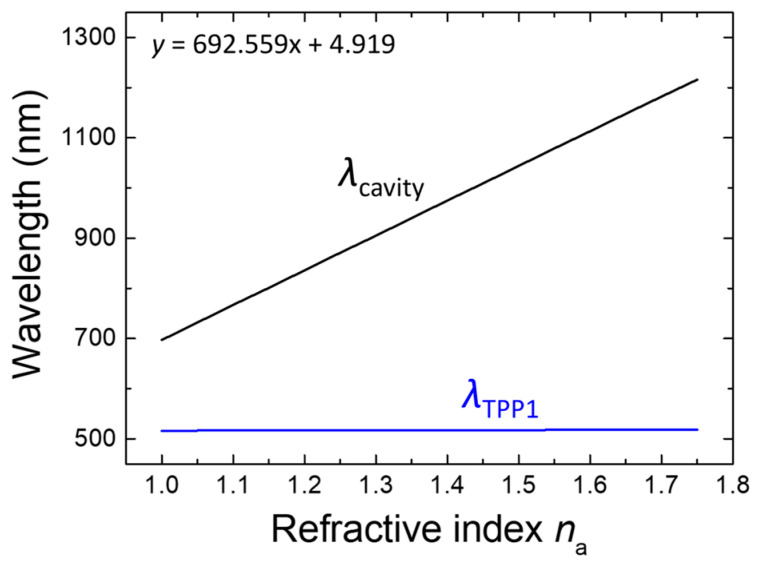
Dependence of the resonance wavelength of the cavity mode on the analyte’s refractive index in the range between 1.00 and 1.75.

To manifest the feasibility of further enhancing the sensing performance based on FoM, a proof-of-concept study was conducted by adjusting the layer thicknesses *d*_H1_ = 47 nm, *d*_L1_ = 60 nm for PC1’s (LH)6H multilayer, *d*_H2_ = 120 nm, *d*_L2_ = 200 nm, and *d*_M_ = 60 nm. This alternative structural scheme yielded a dramatic increase in spectral sharpness, resulting in a remarkably higher FoM. The results of the numerical simulations summarized in Table 3 confirm that the modifications slightly enhance the sensitivity while significantly promoting FoM by more than 80% (from 500–504 to 906–935), ensuring improved detection resolution within a glucose-relevant refractive index range. As depicted in Figure 8, the modified structure exemplifies an applicable refractive-index span of 1.00–1.75, which was intentionally selected to evaluate the structural robustness under extreme RI variations and to illustrate applicability beyond urinary glucose detection, including potential use in chemical, environmental, or industrial sensing. This extended range is not meant to represent typical biomedical scenarios—in such cases, a narrower RI span would be applied, and resolution optimization within that span would be emphasized, indicating its capacity to detect a broader spectrum of analytes and consequently expanding its potential application scope. The enhanced FoM and desired linearity between the resonance wavelength and the analyte’s refractive index across this broad refractive-index range underscore the versatility of the optimized photonic crystal structure, making it suitable for diverse sensing environments and extending its utility to biochemical and environmental monitoring applications. However, this suitability is presently supported only by theoretical simulations, and its realization in practical biosensing would require addressing several critical factors. These include surface fouling, biomolecule immobilization, surface stability, and measurement reproducibility, which remain beyond the scope of this work but are essential for reliable operation under real-world conditions. While such a broad refractive index range may exceed typical biomedical values, it contributes to the structural robustness and highlights the sensor’s potential utility in more diverse or harsh environments. Notably, combining high spectral resolution with an extended RI span has been demonstrated in prior refractive index sensing studies, such as the i-HFFPI fiber sensor, which reliably covers a range from 1.000 to 1.733 without sacrificing signal clarity [47]. This reinforces the importance of developing biosensors with both precision and dynamic range for real-world applications. All resonance shift results in this work are obtained under idealized simulation conditions and are intended to illustrate structural sensitivity rather than experimentally validated performance.

## 4. Conclusions

We introduce an innovative asymmetric photonic structure that combines TPP modes and a cavity mode for passively tunable, highly sensitive optical properties. This design generates hybrid resonances, enhancing spectral selectivity, field confinement, and overall device performance, making it a robust platform for high-precision optical sensing. Using literature-derived refractive indices for human urine as a simulated sample matrix, we evaluated the PC1-M-A-M-PC2 structure’s angular-dependent reflectance spectra, refractive-index responsiveness, and dual-spectrum scheme, assessing its simulated response to glucose-relevant refractive index changes corresponding to higher glucose concentrations.

The TPP1 (TPP2) mode exhibits a stable resonance wavelength, unaffected by changes in the analyte refractive index (*n*_a_), serving as a reliable reference for reflectance- (transmittance-)based sensing. Conversely, the cavity mode shows a linear redshift with increasing *n*_a_, demonstrating high sensitivity. These characteristics enable the devised framework to integrate the fixed TPP1 or TPP2 mode as a reference and the red-shifting cavity mode as a probe, allowing for accurate assessment of subtle refractive index variations, such as those associated with glucose-related refractive index changes in urine.

The device supports both reflectance- and transmittance-based sensing and permits simultaneous or independent spectral analyses across various optical applications. Its angular tunability provides control over resonance wavelengths, facilitating advanced functions such as optical filtering, angle-resolved sensing, and wavelength-selective devices. Key metrics—*Q*-factor, *G*-factor, sensitivity (*S*), and figure of merit (FoM)—were calculated to assess sensing performance. The *Q*-factor measures spectral resolution, whereas the novel *G*-factor introduced in this study balances signal intensity and spectral sharpness. *S* quantifies resonance shifts per *n*_a_ change, and FoM integrates sensitivity and sharpness for holistic analysis.

To contextualize the proposed structure’s sensing capability, we compared its performance metrics with selected refractometric sensors. Juneau-Fecteau et al. [39] demonstrated a Tamm plasmon sensor composed of porous silicon layers transferred onto a gold substrate, achieving a *S* of 139 nm·RIU^−1^ and a *Q*-factor of 25. Meanwhile, Ghayoor et al. [40] designed a high-sensitivity SPR sensor incorporating nanostructured films and graphene, yielding a FoM of 635 RIU^−1^. Compared to these designs, our structure achieves significantly higher sensitivity and FoM, while also offering dual-mode referencing, angular tunability, and an innovative *G*-factor metric, positioning it as a high-performance and versatile platform for refractive index sensing.

This asymmetric photonic configuration conflates stable reference modes and a responsive probe, offering reliability and precision in demanding sensing scenarios. Its innovative design, angular tunability, and compatibility with both spectral modes position it as a versatile candidate for refractive-index sensing and medical diagnostics, as well as various other applications such as chemical sensing, environmental monitoring, and food quality control. Beyond the reported numerical simulation results, experimental studies have been planned to further validate our findings. While the proposed structure exhibits excellent refractive index sensitivity and spectral performance in simulations, its potential specificity toward glucose in complex urine matrices remains to be theoretically assessed and experimentally validated in future work. Since other solutes, such as urea or creatinine, may also influence the refractive index, future experimental work will need to address selectivity, possibly through biochemical functionalization or interference analysis. While the proposed photonic structure demonstrates strong refractive index sensitivity in simulations, translating this design into a practical biosensor will require overcoming several engineering and biochemical challenges. First, microcavity dimensions and fluid handling methods must be optimized to ensure efficient sample introduction and consistent optical interaction. Second, surface functionalization strategies, such as immobilization of biorecognition elements, are necessary to achieve molecular specificity. Third, complex sample matrices like urine may require pre-filtration or conditioning to reduce background interference. Finally, long-term stability, reproducibility, and resistance to surface fouling must be established to support reliable operation in real-world settings. These considerations underscore the role of the present work as a theoretical design platform that can guide future experimental implementation. Future work will focus on optimizing integration into lab-on-a-chip systems, refining mass production techniques, and expanding the simulated sensing framework beyond glucose for broader potential applications. Additionally, hybrid designs incorporating additional photonic or plasmonic elements may pave the way for next-generation sensors, facilitating the transition from laboratory research to practical deployment in healthcare, environmental monitoring, and other fields.

## Figures and Tables

**Table 1:** *Q*- and *G*-factors for different metallic materials used in the TPP-based device. The table summarizes the *Q* and *G* values of the cavity mode for Ag, Au, and Cu in the condition of *n*_a_ = 1.335.

Metallic Materials	*λ*_cavity_ (nm)	*Q*-Factor	*G*-Factor (μm^−1^)
Ag	931.129	718.842	756.675
Au	938.009	189.288	184.334
Cu	938.339	135.252	108.323

**Table 2:** Sensing parameters for the proposed photonic biosensor simulated over refractive indices corresponding to varying glucose concentrations in human urine (*c*). The table presents the corresponding refractive index *n*_a_, TPP1 resonance wavelength *λ*_TPP1_, cavity resonance wavelength *λ*_cavity_, TPP2 resonance wavelength *λ*_TPP2_, sensitivity *S*, and figure of merit FoM. The stable TPP1 (TPP2) mode serves as a reference for the spectral measurement of reflection (transmission), whereas the cavity mode exhibits redshift with increasing *c*, demonstrating the biosensor’s high sensitivity and reliability.

*c*	*n*_a_	*λ*_TPP1_ (nm)	*λ*_cavity_ (nm)	*λ*_TPP2_ (nm)	*S* (nm·RIU^−1^)	FoM (RIU^−1^)
0–15 mg·dL^−1^	1.335	805.397	931.129	1030.456	–	–
0.625 g·dL^−1^	1.336	805.397	931.780	1030.456	650.840	500.261
1.25 g·dL^−1^	1.337	805.397	932.432	1030.456	651.740	500.953
2.5 g·dL^−1^	1.338	805.397	933.084	1030.456	652.660	501.660
5 g·dL^−1^	1.341	805.397	933.738	1030.456	655.410	503.774

**Table 3:** Sensing parameters for the enhanced photonic biosensor at various refractive indices of the analyte. The refined design enhances sensing performance as indicated by improved FoM through structural adjustment.

*n*_a_	*λ*_cavity_ (nm)	*S* (nm·RIU^−1^)	FoM (RIU^−1^)
1.335	929.628	–	–
1.336	930.321	693	905.882
1.337	931.014	693	913.044
1.338	931.707	693	919.098
1.341	933.785	692.833	934.998

## Data Availability

The original contributions presented in this study are included in the article/Supplementary Material. Further inquiries can be directed to the corresponding author.

## Supplementary Materials

The following supporting information can be downloaded at https://www.mdpi.com/article/10.3390/bios15100644/s1, Figure S1: The *Q*-factor and *G*-factor as the device performance metrics; Table S1: Comparative performance of three distinct metals used.
