# Supplementary material for: Simulation of an Asymmetric Photonic Structure Integrating Tamm Plasmon Polariton Modes and a Cavity Mode for Potential Urinary Glucose Sensing via Refractive Index Shifts
Source: Biosensors (Basel). 2025 Sep 29;15(10):644. doi: 10.3390/bios15100644 (PMC12562746; doi:10.3390/bios15100644)
Supplement: Supplementary file 1 [file biosensors-15-00644-s001.zip › biosensors-3753607-supplementary.pdf]

Article

Simulation of An Asymmetric Photonic Structure Integrating Tamm plasmon Polariton Modes and a Cavity Mode for Potential Urinary Glucose Sensing via Refractive Index Shifts

Hung-Che Chou [1], Rashid G. Bikbaev [2], Ivan V. Timofeev [2], Mon-Juan Lee [3] and Wei Lee [4,*]

[1] Institute of Lighting and Energy Photonics, College of Photonics, National Yang Ming Chiao Tung University, Guiren Dist., Tainan 711010, Taiwan; roydinchow3@gmail.com

[2] Kirensky Institute of Physics, Federal Research Center KSC SB RAS, Krasnoyarsk, 660036, Russia; Siberian Federal University, Krasnoyarsk, 660041, Russia; bikbaev@iph.krasn.ru (R.G.B.)

[3] Department of Chemical and Materials Engineering, National Kaohsiung University of Science and Technology, Sanmin Dist., Kaohsiung 807618, Taiwan; mjlee@nkust.edu.tw

[4] Institute of Imaging and Biomedical Photonics, College of Photonics, National Yang Ming Chiao Tung University, Guiren Dist., Tainan 711010; Taiwan

* Correspondence: Wei.Lee@nycu.edu.tw

Supplementary Materials

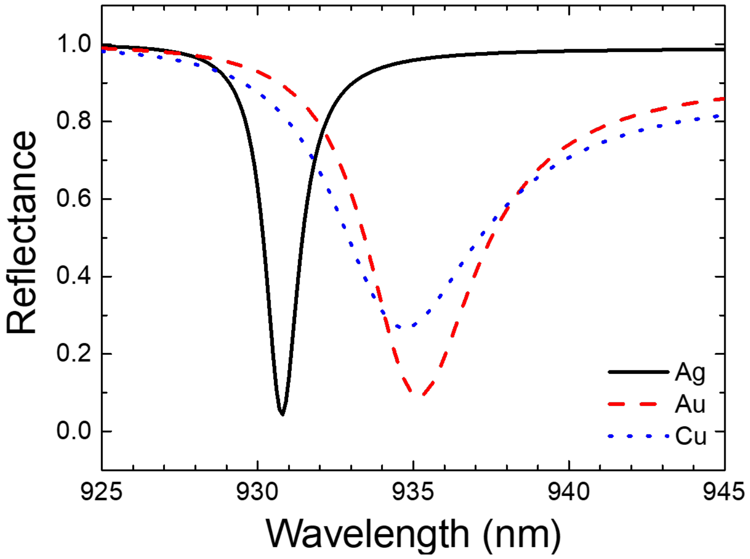

Figure S1. The *Q*-factor and *G*-factor as the device performance metrics. This figure illustrates the reflectance spectra of the cavity mode in a photonic crystal structure individually incorporating three different metallic materials: Ag, Au, and Cu. The accompanying table below presents the corresponding performance metrics—quality factor (*Q*) and guidance factor (*G*)—calculated for each metal. The *Q*-factor characterizes the spectral resolution of the resonance, while the *G*-factor reflects the trade-off between signal intensity and spectral sharpness. A comparative analysis reveals that silver exhibits the highest *Q* and *G* values among the three metals, demonstrating its superior ability to support sharp and well-confined resonances.

$$Q = \frac{\lambda_{cavity}}{\text{FWHM}} \quad (1)$$

$$G = \frac{1 - \text{R}}{\text{FWHM}} \quad (2)$$

Table S1. Comparative performance of three distinct metals used.

**Metallic material**	λ_{cavity}**(nm)**	**FWHM (nm)**	Q**-factor**	**G-factor (μm^{-1})**
Ag	929.87	0.87	1068.82	753.69
Au	934.49	4.20	222.50	86.97
Cu	933.95	7.09	131.73	37.24
